# Mechanism analysis and intervention strategies of the inflammatory microenvironment in traumatic spinal cord injury

**DOI:** 10.3389/fimmu.2025.1692346

**Published:** 2025-12-18

**Authors:** Hou-yun Gu, Fei-xiang Lin, Ning Liu, Can-hua Xu

**Affiliations:** 1 Department of Spine Surgery, Ganzhou Hospital-Nanfang Hospital, Southern Medical University (Ganzhou People’s Hospital), Ganzhou, Jiangxi, China; 2 Department of Spine Surgery, The Affiliated Ganzhou Hospital, Jiangxi Medical College, Nanchang University, Ganzhou, Jiangxi, China

**Keywords:** traumatic spinal cord injury, inflammatory microenvironment, cytokines, chemokines, signal transduction

## Abstract

Traumatic spinal cord injury (tSCI) is a severe disabling central nervous system injury caused by external forces directly acting on the spinal cord. It can rapidly trigger the release of a large number of pro-inflammatory mediators after the injury, leading to significant neurological dysfunction and, in severe cases, paralysis. Currently, symptoms are mainly alleviated, and endogenous repair mechanisms are improved through drug intervention, surgery, stem cell transplantation, behavioral interventions, physical stimulation, and supportive therapies. However, these methods do not directly promote nerve regeneration and functional recovery. The inflammatory response after injury is an important pathological process leading to secondary damage and plays a crucial role in regulating the pathological progression of acute and chronic tSCI. However, prolonged inflammatory stimulation can further worsen the microenvironment at the injury site, leading to neurological function decline. Therefore, regulating the inflammatory microenvironment and restoring cytokine balance are expected to promote the recovery of neurological function after injury. This review summarizes the formation of the inflammatory microenvironment after tSCI, focusing on the recruitment and activation characteristics of major inflammation-related cells, and elaborates on the expression regulation, pathological effects, and impacts of key cytokines—including the interleukin family, TNF-α, and various chemokines—on neuronal survival and axonal regeneration. Additionally, we summarize multiple inflammatory signaling pathways closely related to secondary injury, such as NF-κB, JAK/STAT, and MAPK, emphasizing that these pathways are interconnected. For example, TNF-α and IL-1β can jointly activate NF-κB and MAPK to amplify the pro-inflammatory response and disrupt the blood-spinal cord barrier. Meanwhile, JAK-STAT3 amplifies inflammation while driving reactive proliferation of astrocytes and glial scar formation, thereby limiting later axonal regeneration. Based on this mutually amplifying inflammatory network, we also briefly summarize the exploratory applications of chemical antagonists, biologic agents, neuroprotective molecules, plant-derived active compounds, and hormonal interventions in regulating this microenvironment. This article aims to provide a reference for the analysis of inflammation-mediated pathological mechanisms after tSCI and the development of targeted anti-inflammatory treatment strategies.

## 1 Introduction

Traumatic spinal cord injury (tSCI) is a severe neurological injury caused by external forces (such as impact, falls, or compression) directly acting on the spinal cord. It often results in long-term or even permanent loss of motor, sensory, and autonomic function. The pathological processes of tSCI can be divided into distinct stages, including primary mechanical injury and subsequent secondary injury, with the primary mechanical injury initiating the secondary injury. The latter can trigger a series of secondary pathological changes, such as neuroinflammation, cell death, and limited nerve regeneration (1, 2). Because of the complex pathophysiological processes of tSCI and the very weak neural repair capacity, the damaged spinal cord is difficult to repair and to reconstruct functionally (3, 4), posing significant challenges for healthcare workers and medical research (5, 6). In this context, current clinical treatment strategies mainly focus on the early stage of injury, aiming to preserve residual neurological function as much as possible and to limit further deterioration. Common methods include surgical decompression and spinal stabilization during the acute phase, high-dose corticosteroid therapy, pharmacological interventions, and later rehabilitation training (7). Although some progress has been made in the treatment of tSCI, these conventional treatments can only improve symptoms in the short term and are difficult to prevent long-term neurological deficits and disabilities, leading survivors to still face severe motor, sensory, and autonomic dysfunction for a long time (8). Therefore, in recent years, treatment concepts have gradually shifted from purely mechanical decompression and supportive therapy to interventions targeting the secondary damage process itself, especially focusing on inhibiting the persistent tissue destruction caused by secondary inflammation. Current clinical consensus also suggests that, in addition to implementing spinal decompression as early as possible after injury, efforts should be made to prevent and limit the progression of secondary damage (9). Meanwhile, relevant *in vivo* and *in vitro* studies indicate that neutralizing the local inflammatory microenvironment and inhibiting excessive inflammatory responses may create more favorable regenerative conditions for axonal preservation, myelin protection, and subsequent tissue engineering repair (10). Therefore, a deeper understanding of the inflammatory mechanisms during the secondary damage phase is becoming a key entry point for optimizing tSCI treatment strategies and improving functional recovery.

The inflammatory microenvironment shaped by pro-inflammatory enzymes, inflammatory cells, inflammatory mediators, and other inflammation-related molecules is an important driving force for the occurrence and progression of various central nervous system diseases, including tSCI (11). In tSCI, this inflammatory microenvironment is considered one of the core driving factors of secondary injury, which can directly or indirectly determine the degree of tissue preservation and functional outcomes after injury (12, 13). Following the initial mechanical injury, immune cells such as neutrophils, infiltrating monocytes and macrophages, and T lymphocytes accumulate and become activated at the injury site. At the same time, microglia and astrocytes transform into reactive phenotypes (e.g., M1-like or A1-like states), initiating and amplifying the inflammatory response. This response affects the survival and function of surrounding neurons and creates a microenvironment that inhibits nerve regeneration (14, 15). Multiple *in vivo* and *in vitro* studies have shown that, in the inflammatory microenvironment after tSCI, specific cellular factors such as TNF-α, IL-1β, and IL-6, along with chemokines like CCL2 and CXCL10, drive the inflammatory cascade, disrupt the blood-spinal cord barrier, and promote neuronal apoptosis by regulating signaling between microglia, infiltrating macrophages, and T lymphocytes. These processes thereby influence the balance between nerve injury and repair (16, 17).

It is worth noting that the inflammatory microenvironment after tSCI has a typical ‘double-edged sword’ effect. In the early stages, a moderate inflammatory response clears necrotic tissue and myelin debris, creating conditions for local tissue stabilization and initial repair. When the inflammatory response is continuously amplified through multiple key signaling pathways, it shifts to a pathological and destructive secondary injury process. This amplification is driven by several signaling pathways. The NF-κB pathway, when activated in microglia and infiltrating macrophages, continuously induces the expression of pro-inflammatory factors such as TNF-α, IL-1β, and IL-6. The NLRP3 inflammasome promotes the maturation and release of IL-1β and triggers pyroptosis. The JAK/STAT pathway regulates macrophages and microglia to maintain a pro-inflammatory phenotype during the acute phase or shift to a neuroprotective or repair phenotype in the chronic phase. The MAPK pathway (p38 MAPK, ERK, JNK) is associated with oxidative stress and apoptosis, driving neuronal loss, axonal breakage, and oligodendrocyte death. The sustained activation of these signals not only induces pathological cell death such as apoptosis, pyroptosis, and ferroptosis but also drives reactive astrogliosis, leading to the formation of dense glial scars. These glial scars inhibit axonal regeneration and neural circuit reconstruction during the chronic phase(beyond 28 days after injury), thereby limiting functional recovery (18–20).

Inflammatory signals related to secondary injury can spread along the white matter tracts to adjacent segments, leading to more widespread neuronal death, synaptic connection loss, and long-term impairment of conduction pathways. In the chronic phase, residual activated microglia continuously release pro-inflammatory mediators, sustaining the activation of pathways such as NF-κB, JAK/STAT, and MAPK at a low but persistent level, thereby maintaining a chronic, low-level inflammatory environment. This chronic inflammation not only continues to inhibit axonal regeneration and remyelination but is also closely associated with neuropathic pain, spasticity, and limited recovery of sensory and motor functions (21, 22). Therefore, the inflammatory microenvironment after tSCI should be regarded as a dynamic network shaped by various immune cells (neutrophils, macrophages/microglia, T cells, reactive astrocytes) and key signaling pathways (NF-κB, JAK/STAT, MAPK). The sustained activation of this network is the core driving force behind the expansion of secondary injury, neuronal death, and glial scar formation, as well as the key molecular basis for the limitation of axonal regeneration and functional recovery. Therefore, in-depth analysis of how these signaling pathways coordinate and maintain chronic inflammation over time is crucial for elucidating the pathological mechanisms of tSCI and designing more precise, staged anti-inflammatory treatment strategies.

This narrative review primarily conducts a comprehensive analysis based on representative literature from recent studies on animal models, cellular experiments, and early-stage clinical observational studies in the field of tSCI and related neuroinflammation. This article focuses on and attempts to answer the following core questions: (1) How is the inflammatory microenvironment formed after tSCI, specifically which cell types (such as neutrophils, macrophages and microglia, astrocytes, and T/B lymphocytes) are recruited and activated. (2) How do the cytokines, chemokines, and other mediators released by these cells drive secondary injury through key signaling pathways (such as NF-κB, JAK/STAT, MAPK, etc.)? Specifically, how do these pathways lead to neuronal death, demyelination, and glial scar formation. (3) What intervention strategies are currently available to modulate this inflammatory microenvironment (such as cytokine/receptor antagonists, immunomodulatory drugs, hormones, biological agents, natural products, etc.). What are their action targets and potential translational value. By organizing around these questions, our goal is to systematically elucidate the composition, molecular regulatory mechanisms, and pathological consequences of the inflammatory microenvironment after tSCI. Additionally, we aim to summarize existing and candidate anti-inflammatory/immunomodulatory therapeutic approaches, thereby providing a theoretical basis and direction for future basic research design and clinical intervention strategies.

## 2 Composition and characteristics of inflammatory microenvironment

**Figure 1:**
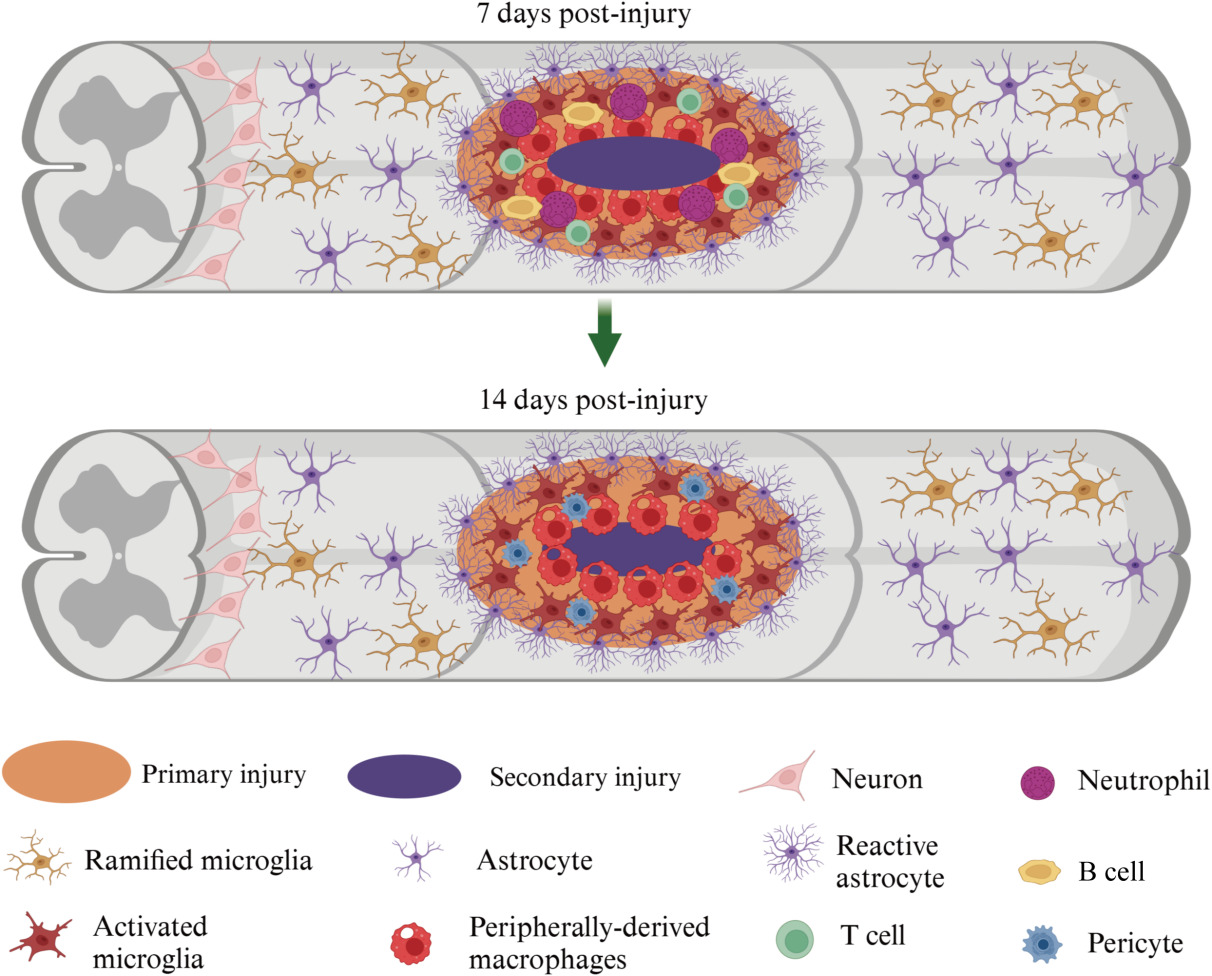
Spatiotemporal cellular changes in the injured spinal cord at 7 and 14 days post-injury. The schematic illustrates the evolution of the lesion microenvironment in the spinal cord following injury, comparing the situation at 7 days post-injury (top panel) and 14 days post-injury (bottom panel). The central lesion core is composed of the primary injury site (orange) surrounded by a region of secondary injury (purple), characterized by progressive inflammatory cell infiltration and glial activation. By 7 days post-injury, the lesion core contains abundant infiltrating peripherally derived macrophages (red), neutrophils (purple), lymphocytes (yellow), and T cells (green), together with pericytes (blue) and reactive astrocytes (violet, stellate with hypertrophic processes) forming a dense inflammatory milieu. Activated microglia (dark red) accumulate around the lesion border, while ramified/resting microglia (brown) and neurons (light pink) remain in the relatively preserved parenchyma distal to the lesion. Astrocytes (light purple) at the periphery of the lesion show marked reactive changes and begin to contribute to glial scar formation. By 14 days post-injury, the lesion core becomes more compact and organized. Peripherally derived macrophages remain prominent, but the relative composition shifts toward a more macrophage-dominated environment with reduced neutrophil presence, indicating a transition from acute to subacute/chronic inflammation. Reactive astrocytes more clearly delineate the lesion border, suggesting maturation of the astroglial scar. Microglia in adjacent tissue persist in an activated state near the injury core, while more distal regions continue to display ramified microglia and relatively preserved neural architecture. (This figure was adapted from Hellenbrand et al., Journal of Neuroinflammation, 2021, (https://doi.org/10.1186/s12974-021-02337-2)).

The inflammatory response after tSCI follows a characteristic temporal sequence, which can be summarized into three stages: acute phase (minutes to hours after injury), early amplification phase (approximately 1–3 days), and sustained amplification and expansion phase (subsequent days to the subacute stage (from approximately 3 days to 14 or 28 days after injury)). Immediately after injury, the blood-spinal cord barrier (BSCB) is disrupted, exposing spinal cord tissue to the circulatory system, and peripheral immune cells (neutrophils, monocytes/macrophages, activated T lymphocytes) begin entering the injury area (23, 24). At the same time, local cells (microglia, astrocytes, endothelial cells, etc.) rapidly release pro-inflammatory cytokines such as TNF-α and IL-1β, along with chemokines including CCL2, CXCL1, and CXCL10, establishing a chemotactic gradient that drives cell recruitment (23, 24). Within 1–3 days, neutrophils and monocyte-derived macrophages infiltrating the spinal cord parenchyma are further activated, becoming the main source of inflammatory mediators such as TNF-α, IL-1β, IL-6, CCL2, and CXCL10 (25). Meanwhile, microglia and astrocytes transform into pro-inflammatory phenotypes and produce the same pro-inflammatory cytokines and chemokines, further amplifying the local inflammatory response (26). In the following days, activated microglia, macrophages, neutrophils, astrocytes, as well as recruited B cells and T lymphocytes continuously release inflammatory mediators, forming a positive feedback loop (27). These signals spread to areas adjacent to the injury, leading to sustained disruption of the BSCB, neuronal death, demyelination, and axonal rupture, thereby promoting the expansion of secondary injury (28, 29)(**Figure 1**).

### 2.1 Infiltration and activation of inflammatory cells

After tSCI, the recruitment and activation of inflammatory cells are core features in the formation of the inflammatory microenvironment and exhibit clear temporal characteristics. Neutrophils are among the first inflammatory cells to arrive at the site of tSCI. Within hours after the injury, neutrophils rapidly migrate from the peripheral blood into the damaged spinal cord tissue, driven by chemokine signals (30, 31). Early neutrophils can clear necrotic cell debris and potential pathogens, providing an acute defense response (32). However, neutrophils also release cytotoxic molecules such as myeloperoxidase (MPO), elastase, and reactive oxygen species (ROS) and form neutrophil extracellular traps (NETs). These molecules and structures can directly damage surrounding neurons, disrupt oligodendrocytes, and compromise the stability of the myelin sheath, thereby exacerbating tissue edema, demyelination, and deterioration of motor function (33, 34).

Microglia, as the innate immune cells in the CNS, maintain the homeostasis of the spinal cord environment under physiological conditions (35). After tSCI, microglia are rapidly activated in the early stage of injury (within the first few hours to 1–3 days after injury), with their morphology and phenotype shifting from a resting state to a pro-inflammatory state. Activated microglia can exhibit functionally polarized phenotypes that resemble the ‘M1 type’ and ‘M2 type’ microglia. M1-type microglia display pro-inflammatory and neurotoxic characteristics, producing a large amount of neurotoxic mediators such as NO and ROS, as well as pro-inflammatory mediators such as TNF-α and IL-1β. These molecules can amplify the local inflammatory cascade response by activating inflammatory signaling pathways such as NF-κB, inducing neuronal apoptosis and exacerbating axonal/neurotrophic damage (36). In contrast, M2-type microglia tend toward an anti-inflammatory/repair phenotype. They participate in limiting excessive inflammation. Additionally, they support tissue remodeling and axonal survival by releasing IL-10 and transforming growth factor-β (TGF-β) and clearing cellular debris (37, 38). Moreover, in a mouse contusion model, it was found that damage-associated molecular patterns (DAMPs) and inflammatory mediators released at the injury site can activate microglia, altering their morphology and function (39, 40). Subsequently, activated microglia can also release cytokines such as IL-1α and TNF-α, which can activate neurotoxic reactive astrocytes, further mediating neuronal death (41). If microglia remain excessively activated and maintain an M1-type pro-inflammatory state, it can lead to chronic low-grade inflammation, resulting in persistent axonal degenerative damage and limited functional recovery (42).

Macrophages mainly derive from peripheral blood monocytes that are recruited and subsequently differentiate after reaching the injury site. In mouse thoracic contusion models and rat compression models, it was found that in the early stages of injury (within hours up to 3 days), chemokines such as CCL2 and CXCL12 in the injury area were significantly upregulated. These chemokines recruit peripheral monocytes into the spinal cord through the CCR2/CXCR4 axis, where they differentiate locally into tissue-infiltrating macrophages (43, 44). These macrophages exhibit high plasticity and can polarize into M1 or M2 phenotypes depending on their microenvironment (30). M1 macrophages, also known as classically activated macrophages, are produced in response to stimuli such as interferon-γ (IFN-γ) and lipopolysaccharides (LPS) (45, 46). In mouse compression models, M1 macrophages were found to have a strong pro-inflammatory effect, capable of secreting large amounts of inflammatory mediators such as TNF-α, IL-1β, IL-6, and nitric oxide (NO). These inflammatory mediators can directly damage neurons and glial cells, disrupt the blood-spinal cord barrier, and exacerbate inflammatory responses and tissue edema (47). Additionally, M1 macrophages can induce neuronal apoptosis or necrosis directly by releasing toxic substances such as ROS and reactive nitrogen species (RNS). They also damage the blood-spinal cord barrier, aggravate local edema, and harm oligodendrocytes, thereby promoting secondary demyelination (48, 49). In contrast, M2 macrophages, also known as alternatively activated macrophages, are produced in response to stimuli such as IL-4 and IL-13. M2 macrophages have anti-inflammatory and pro-repair effects and secrete anti-inflammatory factors such as IL-10 and TGF-β. These factors inhibit inflammatory responses, promote tissue repair, and facilitate nerve regeneration (49, 50). Moreover, M2 macrophages phagocytose and clear cellular debris and pathogens at the injury site, thereby creating a microenvironment conducive to nerve regeneration (50, 51). Therefore, the dynamic balance between M1 and M2 macrophages after injury is considered a key factor in determining the extent of secondary injury and the quality of neurological recovery.

T cells and B cells also participate in the persistent inflammation and immune regulation after tSCI. T lymphocytes are activated after tSCI and enter the injured spinal cord. They play an important role in secondary neuroinflammation, neurodegenerative changes, and repair responses (52). After injury, the balance between pro-inflammatory T cell subsets, such as Th1 and Th17, and regulatory T cells (Tregs) is disrupted. This disruption leads to a Th1/Th17-dominated pro-inflammatory state, which is associated with elevated levels of cytokines such as IFN-γ, TNF-α, and IL-17. These cytokines further amplify focal inflammatory cascades and exacerbate tissue damage (26, 53). These T cell responses subsequently drive the activation of B cells. Both mouse compressive tSCI models and human injured spinal cord specimens show that B cells and plasma cells deposit immunoglobulins in the injury area along with complement deposition. This deposition is associated with local demyelination and axonal rupture (54, 55). In mouse contusion tSCI models, reducing B cells levels in the injured area can improve the subsequent motor function recovery, suggesting that B cell-mediated autoantibody responses contribute to persistent tissue damage (56).

In addition, astrocytes play a dual role in the pathological process after tSCI. On one hand, astrocytes are rapidly activated in response to pro-inflammatory signals (such as TNF-α, IL-1α, and IL-1β). They can acquire a neurotoxic phenotype and secrete additional mediators such as TNF-α, IL-6, and nitric oxide. This secretion further amplifies the local inflammatory response and induces the death of adjacent neurons (57, 58). On the other hand, during the subacute to chronic phase after injury, activated astrocytes form glial scar boundaries and upregulate extracellular matrix components derived from glial cells, such as chondroitin sulfate proteoglycans. These components physically isolate necrotic and high-inflammatory areas, limiting the spread of inflammation to undamaged tissues. Additionally, astrocytes participate in reconstructing the blood-spinal cord barrier, stabilizing the local ionic environment, and supporting the survival of axonal stumps (59). This “neurotoxic/neuroprotective” biphasic characteristic indicates that astrocytes are neither purely harmful nor purely beneficial. Instead, they can exacerbate secondary injury during the acute phase or lay the structural foundation for subsequent tissue stabilization and nerve regeneration during the subacute and chronic phases under varying microenvironmental conditions (60, 61), as summarized in **Table 1**.

**Table 1:** Inflammatory cells involved in the tSCI inflammatory microenvironment.

Cell type	Primary source	Representative secretory molecules /effectors	Main damaging effects	Main protective/ repair effects	References
Neutrophil	Peripheral blood circulation	MPO, elastase, ROS, NETs	Causing damage to neurons and oligodendrocytes; disrupting local tissue structure; amplifying local inflammation; associated with edema and deterioration of motor function	Early involvement in clearing necrotic cell debris and potential pathogens, providing initial defense	(33, 34)
Microglia (M1 type)	CNS resident immune cells	TNF-α, IL-1β, IL-6, NO, ROS; activating NF-κB and other inflammatory pathways	Inducing neuronal apoptosis; exacerbating axonal/neurotrophic damage; driving astrocytes to a neurotoxic reactive type; maintaining chronic inflammation	Mainly a damaging phenotype	(36, 41, 42)
Microglia (M2 type)	CNS resident microglia polarized in specific microenvironments	IL-10, TGF-β; phagocytosing cell debris	Mainly for protective phenotypes	Inhibiting excessive inflammation; supporting the survival of axonal remnants; aiding tissue remodeling; Promoting local homeostasis recovery, limiting the extent of secondary damage	(37, 38)
Macrophages (M1 type)	Differentiated from peripheral monocytes recruited into the spinal cord	TNF-α, IL-1β, IL-6, NO, ROS, RNS	Damaging the blood-spinal cord barrier; exacerbating tissue edema; directly damaging neurons and glial cells; inducing oxidative damage	Mainly a damaging phenotype	(47–49)
Macrophages (M2 type)	Alternative activation phenotype of infiltrating macrophages from peripheral sources	IL-10, TGF-β; phagocytosing cell debris/necrotic remnants	Mainly for protective phenotypes	Inhibiting excessive inflammation; promoting axonal regeneration and remyelination; improving local microenvironment; Promoting tissue repair and functional recovery, regarded as a phenotype favorable for nerve regeneration	(49–51)
T cells (Th1/Th17 pro-inflammatory subgroups)	Adaptive immune cells, peripheral T lymphocytes activated and infiltrated into the spinal cord	IFN-γ, TNF-β, IL-17, etc.	Breaking the balance between Th1/Th17 and Tregs, leading to a pro-inflammatory tendency; amplifying the inflammatory cascade; exacerbating tissue destruction and neurodegenerative changes	Mainly a damaging driver	(26, 53)
T cells (Tregs/Th2 anti-inflammatory subgroups)	Immunoregulatory subgroups among peripheral T cells	IL-10 and other immunosuppressive factors	Inhibiting pro-inflammatory T cell responses, limiting excessive immune attacks	Supporting repair processes, maintaining immune homeostasis, and promote functional recovery	(26, 53)
B cells/Plasma cells	Peripheral B lymphocytes activated and differentiated into plasma cells infiltrating focal areas	Immunoglobulins/auto-antibodies, activating complement	Immune complex and complement deposition associated with demyelination and axonal rupture; related to deterioration of motor function; regarded as a driver of persistent tissue damage	In the current model, protective effects are not significant, and reducing B cell levels may improve motor function recovery	(54, 55)
Astrocytes (neurotoxic reactive type)	Astrocytes in the CNS	TNF-α, IL-6, NO, etc.	Further amplifying local inflammation; inducing death of adjacent neurons; exacerbating secondary damage	Mainly a damaging phenotype	(57, 58)
Astrocytes (repair/barrier type)	Astrocytes in the CNS undergoing reactive remodeling in chronic phases	Gliosis scar matrix components (such as chondroitin sulfate proteoglycans), blood-spinal cord barrier support factors, ion homeostasis regulatory molecules	Forming scar boundaries may inhibit long-range axonal regeneration to some extent	Physically limiting the spread of inflammation; stabilizing the local microenvironment; supporting the survival of axonal remnants, beneficial for subsequent functional preservation	(59)

### 2.2 The role of inflammatory factors and chemokines

Inflammatory cytokines and chemokines play a crucial role in the inflammatory microenvironment after tSCI, interacting through a complex network to participate in the recruitment and activation of inflammatory cells as well as the occurrence and development of nerve damage. TNF-α is a pro-inflammatory cytokine with broad biological activity; it plays a central role in the inflammatory response after tSCI (62). During the acute phase after tSCI (Within several hours to approximately 72 hours after injury), infiltrating macrophages and activated microglia in the CNS rapidly secrete large amounts of TNF-α (63). TNF-α can induce apoptosis in neurons and glial cells by activating the caspase family in a primary neuron and astrocyte co-culture system. This indicates its direct neurotoxic effect (64). Furthermore, in a rat contusion tSCI model, TNF-α was found to increase the permeability of the BSCB, leading to plasma protein extravasation and local edema. At the same time, TNF-α upregulates the expression of IL-1β and IL-6, amplifying the NF-κB-dependent pro-inflammatory cascade (65). Additionally, during the early post-injury phase, the expression level of TNF-α rapidly rises, and the extent of this increase is closely related to the severity of tSCI (66). Higher levels of TNF-α are associated with decreased motor function scores (such as reduced scores on the hind limb motor function scale), enlarged injury volume, increased number of TUNEL-positive cells, and increased extent of axonal rupture (67). Moreover, in mice, blocking TNF-α signaling through gene knockout or administration of TNF-α neutralizing antibodies can reduce secondary tissue damage and improve motor function recovery (68, 69). Therefore, TNF-α is not only an inflammatory marker but also a core mediator and intervention target driving acute secondary damage, including cell apoptosis, barrier disruption, and tissue edema.

Multiple members of the interleukin (IL) family play important roles in the inflammatory microenvironment after tSCI. Specifically, members of the IL family have distinct roles at different stages of tSCI. IL-1β is a potent pro-inflammatory cytokine primarily secreted by activated macrophages and microglia, and its production is closely related to the activation of the NLRP3 inflammasome, which, upon activation, leads to caspase-1 activation that promotes the maturation of pro-IL-1β (70, 71). In a rat compression model, IL-1β activated inflammatory cells, promoted the production of prostaglandin E2 (PGE2) and nitric oxide (NO), and upregulated the expression of cell adhesion molecules, thereby enhancing inflammatory cell infiltration (72). Additionally, IL-1β can directly damage neurons and inhibit the growth and regeneration of nerve axons (73–75). Therefore, IL-1β is considered a key factor linking acute inflammatory amplification with inhibition of axonal regeneration; it plays a dual role in promoting inflammation and suppressing neural regeneration.

IL-6 is a key cytokine that is highly upregulated after tSCI, primarily produced by activated microglia, infiltrating monocyte-derived macrophages, and reactive astrocytes, and is one of the main secretory products of these cells in the injury area (76, 77). In the tSCI mouse contusion model, IL-6 exhibits a phase-dependent dual role: during the acute phase, IL-6 synergizes with TNF-α, and IL-1β to promote immune cell activation and amplify pro-inflammatory signals (78). This process partially relies on the JAK/STAT3 signaling axis, suggesting that IL-6 is not merely a passive bystander but one of the active driving factors maintaining a pro-inflammatory environment. In the subacute and recovery phases, IL-6 regulates immune cells (macrophages and microglia) to transition towards a more reparative phenotype through the JAK/STAT3 pathway, promoting axonal regeneration, glial scar stabilization, and tissue repair and remodeling (79). Therefore, IL-6 is one of the few cytokines that participate in both the amplification of acute injury and the stabilization and repair and remodeling of tissues in later stages, indicating that the JAK/STAT3 axis may be a phase-specific rather than unidirectional therapeutic target. In contrast, IL-10 represents a classic anti-inflammatory pathway regulatory cytokine. It is primarily secreted by M2 macrophages and regulatory T cells (80) and can inhibit the activation of inflammatory cells while reducing the release of pro-inflammatory mediators (TNF-α and IL-1β), thereby exerting anti-inflammatory and neuroprotective effects. Analysis of human tSCI serum samples shows elevated levels of IL-10, which in turn alleviates inflammatory responses and promotes the recovery of neurological function (81). IL-10 is therefore regarded as a key immune regulatory cytokine in the chronic phase that inhibits persistent inflammation, limits further tissue damage, and enhances the preservation of pathway function.

Chemokines are a class of small signaling proteins that direct the migration of inflammatory cells and play a key role in their recruitment (82). In the inflammatory microenvironment after tSCI, the expression of various chemokines undergoes dynamic changes. CCL2 is an important chemokine primarily secreted by activated macrophages, microglia, and endothelial cells (83). CCL2 specifically attracts monocytes, macrophages, and other inflammatory cells to the injury site, promoting their infiltration (84). In an experimental mouse thoracic spinal cord contusion model, during the acute phase after injury, CCL2 expression in the damaged spinal cord tissue is significantly upregulated at the injury site and surrounding areas; this increase is closely related to the degree of infiltration of CCR2^+^ peripheral monocytes/macrophages into the spinal cord parenchyma (85). In the same model, blocking the CCL2-CCR2 axis significantly reduces the recruitment of these peripheral monocyte-derived inflammatory cells to the injury site, thereby inhibiting local inflammatory responses and alleviating secondary tissue damage (86). CXCL12 is another important chemokine that plays a crucial role in nerve regeneration and repair after tSCI (87). After binding to its receptor CXCR4, CXCL12 promotes the migration, proliferation, and differentiation of neural stem cells and progenitor cells, which benefits nerve regeneration (88). However, in the inflammatory microenvironment, CXCL12 can also attract inflammatory cells, such as neutrophils and monocytes, into the injury site, contributing to the amplification of inflammation (89). Therefore, CXCL12 is involved both in the localization of reparative cells and in enhancing inflammation; this dual role reflects its bidirectional nature in tissue reconstruction and inflammation maintenance. CCL20 is also associated with the amplification of neuroinflammation after spinal cord injury. In a rat compressive tSCI model, CCL20 is significantly upregulated in the affected spinal cord tissue after injury and is related to the recruitment of local immune cells, including infiltrating CCR6^+^ lymphocytes and monocyte-derived macrophages, leading to persistent inflammation (90, 91). In the same model, researchers blocked the interaction between CCL20 and its receptor CCR6 by administering neutralizing antibodies against CCL20, resulting in a significant reduction in tissue edema and inflammatory cell infiltration at the injury site, followed by improved recovery of hind limb motor function (92). These results suggest that CCL20 is not merely a passive inflammatory marker, but part of a targetable pro-inflammatory chemokine-receptor signaling axis (**Table 2**).

**Table 2:** Inflammatory cytokines involved in tSCI.

Molecules	Primary source	Cells upregulation timing	Main mechanism of action	Main consequences (damage/protection)	References
TNF-α	Infiltrating monocyte-derived macrophages; activated microglia in CNS	Significantly upregulated during the acute phase (hours to about 72 hours post-injury)	Activates caspase cascade, induces programmed cell death in neurons and astrocytes; increases blood-spinal cord barrier permeability, leading to plasma protein extravasation and edema; amplifies the release of pro-inflammatory factors such as IL-1β and IL-6	Pro-inflammatory, exacerbates secondary tissue damage, worsens neurological function	(65–67)
IL-1β	Activated macrophages and microglia	Rapidly elevated during the acute phase	Promotes the production of prostaglandin E2 (PGE2) and nitric oxide (NO); upregulates adhesion molecule expression, enhancing adhesion and infiltration of peripheral inflammatory cells; can directly damage neurons and inhibit axonal growth	Amplifies local inflammatory response, inhibits regeneration	(73–75)
IL-6	Microglia; infiltrating monocyte-derived macrophages; reactive astrocytes	Highly expressed during the acute phase and sustained into the subacute/recovery phase	Acute phase: promotes activation of inflammatory cells accompanied by upregulation of TNF-α and IL-1β; subacute/recovery phase: activates JAK/STAT3 pathway, driving microglia and macrophages to transition to anti-inflammatory/repair phenotype	Early phase: pro-inflammatory, amplifies secondary damage; later phase: promotes repair, supports axonal regeneration, stabilizes glial scars, and tissue remodeling	(78, 79)
IL-10	M2 macrophages; regulatory T cells (Tregs)	Starts in the subacute phase, detectable elevation in human tSCI serum	Inhibits activation of pro-inflammatory cells (such as M1-like macrophages/activated microglia); downregulates pro-inflammatory mediators like TNF-α and IL-1β	Anti-inflammatory and neuroprotective; associated with better functional recovery	(81)
CCL2	Activated macrophages; microglia; endothelial cells	Significantly upregulated in the acute phase (approximately 0–3 days) in the local injured spinal cord tissue	Attracts peripheral monocytes into the injury area via the CCR2 axis, differentiating into infiltrating macrophages locally	Recruits a large number of inflammatory cells, amplifying local inflammation and secondary tissue damage	(84–86)
CXCL12	Primarily produced by cells in the injured spinal cord area (including glial cells, endothelial cells, etc.)	Sustained expression after the acute phase, continuing into the repair phase	After binding to its receptor CXCR4, promotes migration, proliferation, and differentiation of neural stem/progenitor cells, supporting regeneration; can also attract neutrophils and monocytes, participating in inflammatory cell recruitment	Can promote regeneration (mobilization of neural progenitor cells) and also promote infiltration of inflammatory cells in an inflammatory environment	(88, 89)
CCL20	Affected spinal cord tissue cells (including local immune cells)	Significantly upregulated during the acute phase in a rat compressive tSCI model	Chemotactic for CCR6^+^ immune cells (such as certain lymphocyte subsets and monocyte-derived macrophages) into the injury area, maintaining persistent inflammation; associated with tissue edema and accumulation of inflammatory cells	Pro-inflammatory; associated with deterioration of motor function	(90, 91)

## 3 The impact of inflammatory microenvironment on tSCI

### 3.1 Neurotoxicity

**Figure 2:**
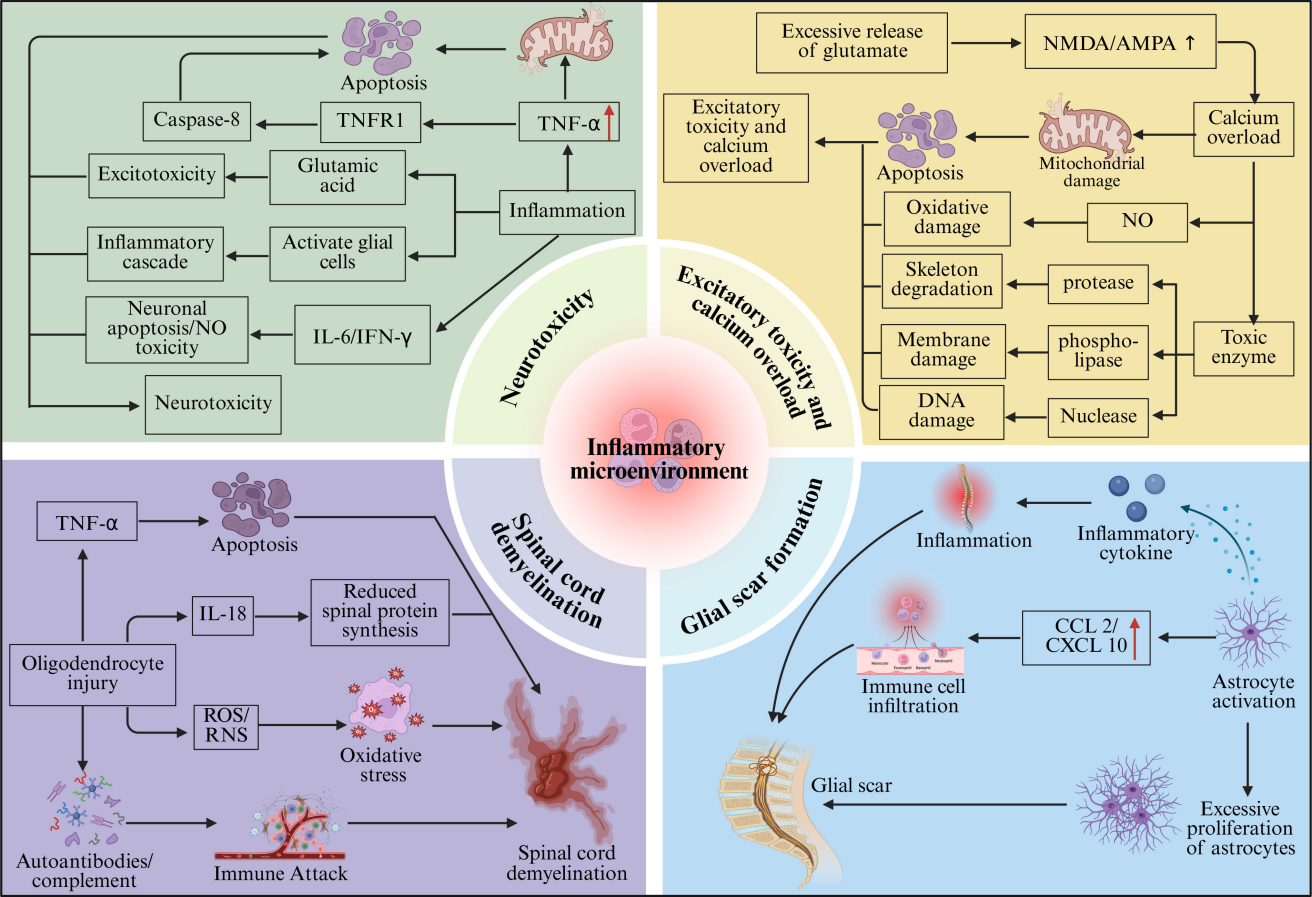
Schematic diagram of the influence of the inflammatory microenvironment on the pathological process of tSCI. The inflammatory microenvironment mainly affects tSCI through neurotoxicity, excitotoxicity and calcium overload, glial scar formation, inhibition of axon growth and spinal cord demyelination.

Inflammatory factors are released in large quantities in the inflammatory microenvironment after tSCI, producing significant neurotoxic effects on neurons, and their damage mechanisms involve multiple aspects. TNF-α, a key pro-inflammatory cytokine, is significantly upregulated within hours after injury in the rat compression injury model. TNF-α can lead to neuronal apoptosis by activating intracellular apoptosis signaling pathways (93). Specifically, after TNF-α binds to TNF receptor 1 (TNFR1) on the neuronal membrane surface, it first recruits the adaptor protein TRADD containing a death domain, and then further recruits Fas-associated death domain protein (FADD) and caspase-8, which assemble to form the death-inducing signaling complex (DISC) (94, 95). This complex mediates the activation of caspase-8. The activated caspase-8 amplifies neuronal damage signals through two pathways: one is the extrinsic (receptor-mediated) apoptotic pathway, where caspase-8 directly cleaves and activates downstream effector caspases (such as caspase-3 and caspase-7), leading to DNA fragmentation, chromatin condensation, and neuronal apoptosis (96). The other pathway is the mitochondrial-dependent intrinsic apoptotic pathway, where caspase-8 cleaves Bid to generate truncated Bid (tBid), which increases mitochondrial outer membrane permeability, leading to the release of cytochrome c, further activating the caspase cascade reaction, thereby amplifying the apoptotic signal (97). In addition to directly inducing apoptosis, TNF-α can also amplify the secondary injury network: it promotes the abnormal accumulation of glutamate at the injury site (exacerbating excitotoxicity) and activates astrocytes, causing them to release pro-inflammatory cytokines such as IL-6 and IFN-γ, thereby driving the inflammatory cascade, ROS/RNS-mediated neuronal damage, and degenerative changes in axons and synapses (98). Overall, this TNF-α/TNFR1–TRADD/FADD–caspase-8 axis not only induces apoptosis in individual neurons but also promotes secondary tissue loss and deterioration of neurological function. Consistent with these findings, in the rat spinal cord contusion model, pharmacological inhibition of TNF-α expression or blockade of the TNF-α signaling pathway can significantly reduce the level of caspase-dependent apoptosis in neurons within the injury area, alleviate secondary neural tissue damage, and improve subsequent motor function recovery (99). In addition to TNF-α, IL-1β has also been proven to be an important neurotoxic factor. Evidence from the rat spinal cord contusion model shows that local elevation of IL-1β disrupts the integrity of the BSCB, increases microvascular permeability, leading to the extravasation of plasma proteins and peripheral inflammatory cells into the injury area, inducing local edema and secondary tissue compression, thereby exacerbating mechanical stress, and functional impairment on remaining neurons (100, 101). Additionally, in an *in vitro* co-culture system of primary spinal cord neurons and glial cells, exogenous IL-1β can reshape neurotransmitter homeostasis. On one hand, it promotes the release of the excitatory neurotransmitter glutamate, while on the other hand, it inhibits the synthesis and release of the inhibitory neurotransmitter gamma-aminobutyric acid (GABA). Excess glutamate can trigger excitotoxicity. Meanwhile, the decrease in GABA weakens the inhibitory regulation of neuronal excitability, and this excitatory–inhibitory imbalance is considered an important molecular basis for secondary neuronal damage (102). In addition to the *in vitro* findings, significant elevation of IL-1β has also been detected in biopsy samples from human injured tissues surrounding the lesion, where IL-1β can activate microglia and astrocytes, prompting them to release more inflammatory mediators such as TNF-α and IL-6, forming an inflammatory cascade that exacerbates neuronal damage (103). This suggests that the aforementioned inflammation–excitotoxicity axis is not limited to rodent models. In addition to TNF-α and IL-1β, inflammatory cytokines such as IL-6 and IFN-γ also contribute to neuronal damage mediated by the inflammatory microenvironment. IL-6 is significantly upregulated during the acute phase in spinal cord contusion models in rats (104). In these animal models and neuronal *in vitro* culture systems, IL-6 binds to the IL-6 receptor complex on the surface of neurons and activates the JAK/STAT3 pathway. This activation induces transcriptional programs associated with apoptosis, inflammatory stress, and axonal dysfunction, which manifest as decreased neuronal survival and impaired axonal conduction (105, 106). In contrast, IFN-γ mainly originates from activated T lymphocytes; a large number of IFN-γ–positive T cells have been observed in mouse spinal cord compression injury models and among infiltrating lymphocytes around human tSCI lesions (107, 108). IFN-γ enhances the pro-inflammatory activity of infiltrating macrophages and resident microglia, increasing their production of highly reactive molecules such as NO. At the same time, IFN-γ can also directly act on neurons, inducing them to produce neurotoxic molecules, including NO, and exacerbating mitochondrial stress, thereby amplifying secondary neuronal damage (109, 110) (**Figure 2**).

### 3.2 Excitatory toxicity and calcium overload

Excitotoxicity refers to the excessive extracellular excitatory neurotransmitter accumulation (mainly glutamate) and the overactivation of neuronal excitatory receptors, which induces pathological ionic influx and cell death in neurons (111, 112). Calcium overload, on the other hand, refers to the abnormal and sustained increase of free Ca^2+^ concentration within neurons to toxic levels, thereby inducing intracellular protein degradation, membrane damage, mitochondrial failure, and apoptosis (113). In the inflammatory microenvironment following tSCI, excitotoxicity and calcium overload are core mechanisms that lead to progressive neuronal death and functional loss. These two processes amplify each other through interactions with inflammatory responses and glial scar formation, ultimately affecting axonal regeneration and functional recovery. They represent sequential upstream and downstream events: glutamate-driven excitotoxicity induces and maintains calcium overload, and calcium overload causes irreversible neuronal structural damage by converting the initial excitotoxic stimulus (114). Under physiological conditions, excitatory and inhibitory neurotransmitters in the spinal cord are in dynamic balance to maintain neuronal firing, synaptic plasticity, and ionic homeostasis (115). However, in the rat tSCI model, the local microenvironment rapidly exhibits pathological accumulation of extracellular glutamate after injury, disrupting this balance. The reasons mainly include two aspects: first, mechanical injury leads to the rupture of neuronal cell membranes, causing passive leakage of high concentrations of intracellular glutamate; at the same time, the inflammatory response further promotes excessive release of glutamate. Activated microglia, neutrophils, and monocyte-derived macrophages infiltrating the injury site can actively release glutamate under the stimulation of pro-inflammatory signals such as TNF-α and IL-1β, thereby continuously amplifying excitotoxicity in the core injury area (116, 117). Second, the ability of astrocytes to clear glutamate significantly decreases after spinal cord injury. Under normal conditions, astrocytes rely on high-affinity glutamate transporters (such as GLT-1/EAAT2) to uptake synaptic glutamate into cells and convert it into glutamine for metabolic’detoxification’via glutamine synthetase (118, 119). However, in the injury area, astrocytes undergo reactive changes and swelling, and the expression and transport activity of GLT-1/EAAT2 are downregulated (120). Meanwhile, local energy crisis (ATP depletion, Na^+^/K^+^ gradient collapse, membrane potential destabilization) weakens the driving force of Na^+^-dependent glutamate transporters, leading to impaired reuptake and disrupted metabolic cycling of glutamate (121). Consequently, glutamate is continuously released and cannot be effectively cleared, causing extracellular glutamate to sharply and persistently increase around the injury site. Glutamate is the main excitatory neurotransmitter in the central nervous system. An appropriate amount of glutamate is crucial for the transmission of neural signals and the normal function of neurons (122). However, when the local glutamate concentration in the spinal cord is excessively elevated, it strongly and persistently activates the ionotropic glutamate receptors on the surface of spinal cord neurons and glial cells. These receptors include the N-methyl-D-aspartate (NMDA) and AMPA receptors (123). Damage-Associated Molecular Patterns (DAMPs) and pro-inflammatory signals can drive astrocytes into a highly reactive state, continuously activating pathways such as JAK/STAT3. This leads to their proliferation, hypertrophy, and increased expression of GFAP at the injury site, ultimately forming a dense glial scar barrier. Furthermore, this excessive activation induces the typical excitotoxic response, where receptor-mediated cation channels remain open for an extended period. This results in a massive influx of Ca^2+^ and Na^+^ into neurons, with the pathological influx of Ca^2+^ considered a key early event in secondary neuronal death (124). It is important to emphasize that calcium overload after tSCI does not solely arise from the opening of NMDA/AMPA receptors themselves. Continuous activation of glutamate receptors leads to significant membrane depolarization and excessive Na^+^ influx, which can trigger further opening of voltage-dependent Ca^2+^ channels and reverse the Na^+^/Ca^2+^ exchanger from a physiological mode of “pumping out Ca^2+^” to a pathological mode of “pumping in Ca^2+^”, thereby continuously pushing Ca^2+^ into the cells (125, 126). In addition, oxidative stress can also promote the release of stored Ca^2+^ from intracellular calcium stores such as the endoplasmic reticulum, further increasing the concentration of free Ca^2+^ in the cytoplasm (127). In summary, calcium overload after tSCI is a complex result driven by excessive activation of glutamate receptors, membrane electrophysiological destabilization, reverse operation of ion transporters, and abnormal leakage of intracellular calcium stores.

Calcium overload has a severe damaging effect on neurons. Under normal circumstances, intracellular Ca^2+^ levels are tightly regulated to remain low and act as controlled signaling molecules involved in intracellular regulation. When Ca^2+^ is abnormally elevated, it activates a series of calcium-dependent enzymes, including proteases, phospholipases, and nucleases (128). The activation of proteases leads to the degradation of cytoskeletal proteins, disrupting the structural integrity of neurons (129). The activation of phospholipases hydrolyzes phospholipids on cell membranes, resulting in damage and dysfunction of the cell membrane (130). The activation of nucleases degrades DNA and RNA, affecting the transmission of genetic information and protein synthesis in cells (131). Meanwhile, excessive Ca^2+^ entering mitochondria interferes with mitochondrial membrane potential and inhibits ATP synthesis. It also induces mitochondria to release cytochrome c and other pro-apoptotic factors, thereby triggering the mitochondrial-dependent apoptosis pathway (132, 133). In addition, excessive Ca^2+^ can induce neurons and activated microglia to upregulate NOS, leading to increased production of NO. In tissue samples around the injury site of the spinal cord compression injury model in mice, the increase of NO is often accompanied by the accumulation of superoxide anion (O_2_·^−^) (113). NO and O_2_·^−^ can rapidly react to form peroxynitrite (ONOO^−^), which is a strong oxidizing and nitrating agent capable of attacking key macromolecules such as proteins, membrane lipids, and nucleic acids, causing irreversible oxidative damage and further amplifying neuronal death signals (134) (**Figure 2**).

### 3.3 Glial scar formation and axonal growth inhibition

Astrocytes are the most abundant glial cells in the central nervous system. After tSCI, various stimuli in the inflammatory microenvironment (including DAMPs and pro-inflammatory cytokines such as TNF-α and IL-1β), rapidly activate astrocytes, inducing their transition from a resting state to a reactive state. Reactive astrocytes show typical morphological and functional remodeling: their cell bodies enlarge, and the number and thickness of astrocytic processes significantly increase. They also begin to express various cytokines, chemokines, and neurotrophic factors at high levels (135, 136). In the injury area, inflammatory cells (such as infiltrating monocyte-macrophage-like cells and T cells) migrate in and release TNF-α, IL-1β, and chemokines CCL2 and CXCL10. These molecules not only continuously amplify the local inflammatory response but also directly promote the proliferation and reactive changes of astrocytes.

During the acute phase of the inflammatory response after tSCI, reactive astrocytes mainly present a pro-inflammatory phenotype. They secrete high levels of TNF-α, IL-1β, and IL-6, which can further activate local microglia and monocyte-derived macrophages infiltrating the injury area, maintaining the sustained activation of pro-inflammatory signaling pathways such as TNF-α and IL-1β, thereby exacerbating the inflammatory response (137). In addition, TNF-α and IL-1β can upregulate the expression of inflammation-related genes by activating the nuclear factor-κB (NF-κB) signaling pathway, promoting the massive release of inflammatory mediators (138). Furthermore, activated astrocytes can also produce chemokines (such as CCL2 and CXCL10), which have been shown in rat tSCI models to recruit peripheral immune cells (including monocyte-macrophage-like cells and T cells) to further infiltrate the injury site, forming a sustained supply of immune cells and enhancing the local inflammatory amplification loop (139). This high-level exposure to pro-inflammatory cytokines and chemokines can have direct cytotoxic effects on surrounding surviving neurons, oligodendrocytes, and non-dead glial cells, inducing apoptosis and necrosis, and are closely related to the limited recovery of motor and sensory functions (140). Therefore, astrocytes at this stage are not only responders driven by inflammation but also amplifiers of the inflammatory cascade.

Under stimulation by continuous inflammatory signals (such as TNF-α, IL-1β, CCL2, CXCL10), astrocytes undergo excessive activation and proliferation, gradually forming a dense glial scar structure at the boundary of the injury site. The glial scar, which is mainly composed of astrocytes and the extracellular matrix they secrete, exhibits significant dual functions (141). On one hand, from the perspective of spatial structure, the cell bodies and thickened processes of activated astrocytes intertwine to form a dense network that wraps and encloses the core area of injury, creating a physical barrier; this barrier can limit the spread of inflammatory cells and cytotoxic molecules to the surrounding, less severely damaged spinal cord tissue, playing an acute protective role of “encapsulation” and “isolation,” thereby reducing further secondary injury (142). On the other hand, from the perspective of regeneration, this glial boundary also hinders axon regeneration. In the rat tSCI model, axon regeneration is significantly restricted within the glial scar formed at the injury site; the disordered fiber orientation further impedes the establishment of orderly and long-range axonal connections necessary for neural circuit reconstruction (143, 144). In addition to the physical barrier, the glial scar also forms a chemical barrier. Reactive astrocytes secrete and enrich various inhibitory extracellular matrix components (such as chondroitin sulfate proteoglycans) during this stage; these molecules can inhibit axonal extension, anchor, and physically trap regenerating nerve fibers, limiting their ability to traverse the injury area for axonal reconstruction (145, 146). Furthermore, astrocytes and accompanying myelin/oligodendrocyte debris can release a series of growth-inhibitory molecules, including Nogo-A (reticulon-4A), myelin-associated glycoprotein (MAG), and oligodendrocyte myelin glycoprotein (OMgp). These inhibitory molecules can bind to corresponding receptors on the surface of neurons, initiating axonal growth inhibition signaling pathways, thereby directly limiting axon regeneration and extension (147, 148). Therefore, the glial scar serves as a protective structure that limits the spread of inflammation and stabilizes tissue boundaries during the acute phase after tSCI, while also acting as a barrier that hinders axon regeneration and obstructs the reconstruction of long-range neural circuits during the chronic phase (**Figure 2**).

### 3.4 Spinal cord demyelination

The main function of oligodendrocytes is to form myelin sheaths that wrap around the axons of neurons, facilitating the rapid conduction of nerve impulses. In the inflammatory microenvironment after tSCI, oligodendrocytes are highly susceptible to damage, leading to apoptosis and functional impairment, which results in myelin loss and severe disruption of nerve conduction (149, 150). This process is not caused by a single pathway but results from the synergistic effects of direct toxic actions mediated by inflammatory factors, oxidative stress-mediated structural damage, and sustained immune attacks mediated by adaptive immunity. Firstly, inflammatory factors can directly induce the death of oligodendrocytes. Moreover, TNF-α is significantly upregulated in the inflammatory microenvironment after tSCI and can directly act on oligodendrocytes. TNF-α binding to TNFR1 on the surface of oligodendrocytes activates intracellular apoptotic signaling pathways, triggering the cascade activation of caspase family proteases, ultimately inducing apoptosis (151, 152). Similarly, pro-inflammatory cytokines such as IL-18 are also believed to promote oligodendrocyte apoptosis and inhibit the synthesis of myelin proteins, driving the occurrence of secondary demyelination. During this process, not only does the number of oligodendrocytes decrease, but the structural proteins necessary for synthesizing and maintaining myelin are also suppressed. IL-1β can damage oligodendrocytes through multiple pathways by inhibiting the proliferation and maturation of oligodendrocytes and reducing the expression of key myelin-related proteins, such as myelin basic protein, MBP, proteolipid protein, PLP, etc., thereby weakening their ability to maintain myelin homeostasis (153). In addition, IL-1β promotes the production of ROS and RNS, leading to oxidative stress damage that disrupts the structure and function of oligodendrocytes (154).

Secondly, the inflammatory microenvironment can trigger significant oxidative/nitrosative stress, causing further damage to oligodendrocytes and myelin structures. In the context of inflammatory cell activation and metabolic abnormalities, a large number of ROS and RNS (such as O_2_^−^, hydrogen peroxide, H_2_O_2_, hydroxyl radical, ·OH, nitric oxide, NO, and peroxynitrite, ONOO^−^) are continuously released. These highly reactive molecules can attack key target structures such as the plasma membrane, mitochondria, and nucleic acids of oligodendrocytes (155, 156). After lipid peroxidation of the membrane lipids, the permeability of the cell membrane increases abnormally, disrupting ionic homeostasis and significantly impairing cell function. Mitochondrial damage leads to restricted energy metabolism and reduced ATP synthesis, which makes it more difficult for cells to maintain myelin synthesis and repair. Additionally, nucleic acid damage can cause genomic instability and induce apoptotic pathways (157). Furthermore, oxidative stress can further activate apoptotic signals such as the mitochondrial pathway and death receptor-mediated pathway, inducing apoptosis in oligodendrocytes (158). Therefore, ROS and RNS are not merely byproducts but are core mediators that promote oligodendrocyte death, myelin damage, and axonal exposure.

Furthermore, the adaptive immune response can form a sustained and directed attack on oligodendrocytes and myelin components. After tSCI, B cells/plasma cells can produce autoantibodies against myelin components and oligodendrocyte antigens (159). After deposition of these autoantibodies in the damaged area, they can activate the complement system, triggering complement-dependent cytotoxic reactions that directly damage oligodendrocytes and myelin sheaths (160). At the same time, autoantibodies can also recruit immune cells such as natural killer (NK) cells and macrophages through antibody-dependent cell-mediated cytotoxicity to mediate cytotoxic attack on oligodendrocytes (161). Additionally, activated T lymphocytes can directly attack oligodendrocytes, exacerbating their apoptosis and shedding. Following oligodendrocyte damage, myelin integrity is compromised, resulting in demyelination. Demyelination can slow down or even interrupt the conduction speed of nerve impulses, severely affecting neurological function (162). In tSCI patients, demyelination is one of the important causes of neurological dysfunction such as limb paralysis and sensory disturbances (163). Axons that lose myelin are also more susceptible to continuous inflammation and oxidative stress, further undergoing degeneration and disruption, forming a vicious cycle of “oligodendrocyte loss – demyelination – secondary axonal injury,” ultimately exacerbating long-term neurological deficits after tSCI (164) (**Figure 2**).

## 4 Inflammatory signaling pathways and molecular regulatory mechanisms

The inflammatory microenvironment after tSCI is a highly dynamic and coordinated network, where multiple signaling pathways jointly regulate immune activation, oxidative stress response, and neural tissue remodeling. The previous section has generally introduced the inflammation and immune imbalance mechanism after tSCI, and this section will further focus on the four core signaling pathways that play a key role in the regulation of inflammation in tSCI—namely, the TLR4/NF-κB pathway, MAPK pathway, JAK/STAT pathway, and PI3K/Akt pathway. These pathways are currently the most commonly reported important molecular mechanisms mediating the inflammatory response and neuroimmune regulation after tSCI in the literature.

### 4.1 NF-κB signaling pathway

NF-κB is a member of the Rel protein family and is an evolutionarily conserved transcription factor (165), involved in the adaptation and response of cells to various environmental changes (166, 167). NF-κB usually exists in an inactive form in the cytoplasm. Upon stimulation by DAMPs, bacterial endotoxins, or pro-inflammatory cytokines (such as TNF-α and IL-1β), IκBα is phosphorylated and degraded. This process allows NF-κB to translocate into the nucleus and initiate transcription programs, inducing the expression of various inflammation-related genes (such as TNF-α, IL-1β, IL-6) as well as chemokines and adhesion molecules (168, 169). After tSCI, the NF-κB signaling pathway is regarded as a key driving factor of secondary inflammatory responses. Studies from rat acute spinal cord injury models show that mechanical injury rapidly leads to the release of a large amount of cellular debris, DAMPs, and pro-inflammatory cytokines, thereby activating infiltrating macrophages, resident microglia, and reactive astrocytes in the injury site and surrounding areas. Among these cells, upstream signals such as cytokines like TNF-α can trigger NF-κB nuclear translocation, thereby amplifying the inflammatory transcription response (170, 171). Activated microglia can secrete large amounts of mediators such as TNF-α, IL-1β, and IL-6 within hours after injury. These mediators, in turn, continuously stimulate the NF-κB pathway, forming a self-amplifying inflammatory loop (172, 173). Meanwhile, NF-κB-dependent transcription also promotes the release of factors such as CXCL10, CCL2, TGF-β2, and adhesion molecules by reactive astrocytes. These molecules can recruit more peripheral immune cells into the injury area, enhance adhesion and infiltration, and promote BSCB disruption, cytotoxic accumulation, and secondary tissue damage, thereby exacerbating neurological deficits after tSCI (174–177). Since NF-κB is located at the upstream hub of “initial injury signals → inflammation amplification → tissue damage,” inhibiting its activation can reduce the expression of mediators such as TNF-α, IL-1β, and IL-6, decrease immune cell infiltration and BSCB disruption, and alleviate secondary neuronal damage. Therefore, NF-κB is considered a potential therapeutic target for controlling excessive inflammation and limiting secondary damage after tSCI.

### 4.2 JAK/STAT signaling pathway

The JAK/STAT signaling pathway plays an important role in the inflammatory response of astrocytes, especially the activation of JAK2 and STAT3 (178). In a rat model of acute spinal cord injury, rapid and sustained phosphorylation of STAT3 in astrocytes can be detected in the injury area and its surrounding region, closely matching the early focal neuroinflammation after injury (179, 180). Similarly, *in vitro* experiments with primary astrocytes exposed to inflammatory stimuli such as bacterial endotoxin LPS or cytokines like TNF-α, IL-1β, and IL-6 rapidly activate JAK2, leading to STAT3 phosphorylation. Phosphorylated STAT3 then translocates to the nucleus, initiating a transcriptional program activating multiple inflammation-related genes, including typical pro-inflammatory cytokines such as TNF-α, IL-6, and IL-1β (181, 182). These mediators, in turn, can exacerbate the activation of local microglia and macrophages as well as neurotoxic stress, thereby amplifying the secondary neuroinflammatory response associated with tSCI (183). Notably, the activation of STAT3 is observed not only in rodent models but also in GFAP-positive reactive astrocytes at the injury margin of severe tSCI patients. In these cells, enhanced nuclear localization of STAT3 and upregulation of its downstream inflammatory factors can also be detected (184).

In addition to mediating the expression of pro-inflammatory cytokines, the JAK2/STAT3 pathway is closely related to the reactive proliferation, morphological remodeling, and scar formation of astrocytes (185). During the subacute phase of the spinal cord contusion/compression injury model in rodents, persistent activation of STAT3 signaling coincides with the dense arrangement of astrocytes at the injury boundary, thickened processes, and high levels of GFAP expression, which together form the typical astrocyte-dominated glial scar (184). This glial scar structurally forms a covering and an isolating barrier around the injury core, while simultaneously creating both a mechanical barrier and a molecularly inhibitory environment for regenerating axons. This environment is believed to limit axonal regeneration and subsequently neural circuit reconstruction, thereby posing a long-term obstacle to functional recovery (186). A similar dense GFAP^+^/STAT3^+^ reactive astrocyte barrier has also been described at the margins of severe human tSCI lesions, correlating with limited functional recovery in the chronic phase (187, 188). Therefore, the JAK2/STAT3 pathway serves as a core signaling module that drives astrocytes to produce pro-inflammatory cytokines. Moreover, it amplifies secondary neuroinflammation and acts as a key regulator, inducing reactive proliferation of astrocytes and glial scar formation, thereby affecting axonal regeneration and functional recovery. Consequently, targeting JAK2/STAT3 signaling may provide a potential therapeutic strategy to limit the harmful inflammatory activation and excessive scar formation of astrocytes after tSCI.

### 4.3 TLR4/NF-κ B pathway

Toll-like receptor 4 (TLR4) is a pattern recognition receptor that plays an important role in the body’s response to infection and injury by mediating pro-inflammatory responses through activation of the NF-κB signaling pathway. In rat spinal cord injury, TLR4 is significantly upregulated in glial cells and infiltrating immune cells (macrophages and neutrophils) in the injury area within hours to days after injury. This upregulation is accompanied by enhanced phosphorylation and nuclear translocation of NF-κB. The activated NF-κB then induces excessive release of pro-inflammatory factors such as TNF-α, IL-1β, and IL-6. These factors not only exacerbate local inflammatory responses, but may also lead to further neuronal damage and apoptosis (189, 190). Furthermore, abnormal activation of TLR4 is closely related to the progression of various neurological diseases. For example, high expression of TLR4 has been observed in human spinal cord injury tissue samples, chronic activation occurs in neurodegenerative disease models, and sustained inflammatory activation is present in chronic pain states (191, 192). In the rat tSCI model, the use of TLR4 inhibitors or NF-κB signaling blockers can significantly reduce inflammatory infiltration and edema in the injury area, reduce levels of pro-inflammatory factors, and improve motor function recovery (193, 194). This indicates that the TLR4/NF-κB pathway plays a key amplifying role in the secondary inflammatory response following tSCI. Additionally, *in vitro* cell culture studies have found that activation of TLR4 can drive microglia to adopt M1 polarization, further exacerbating neuroinflammation. In contrast, inhibiting TLR4 or inhibiting NF-κB activation can enhance the proportion of microglia exhibiting M2 polarization and increase the secretion of anti-inflammatory factors such as IL-10 and TGF-β (195). Therefore, regulating the balance of the TLR4/NF-κB signaling axis and microglial polarization state may become a potential therapeutic strategy to alleviate neuroinflammation and promote neurological recovery after tSCI.

### 4.4 Mitogen activated protein kinase

**Figure 3:**
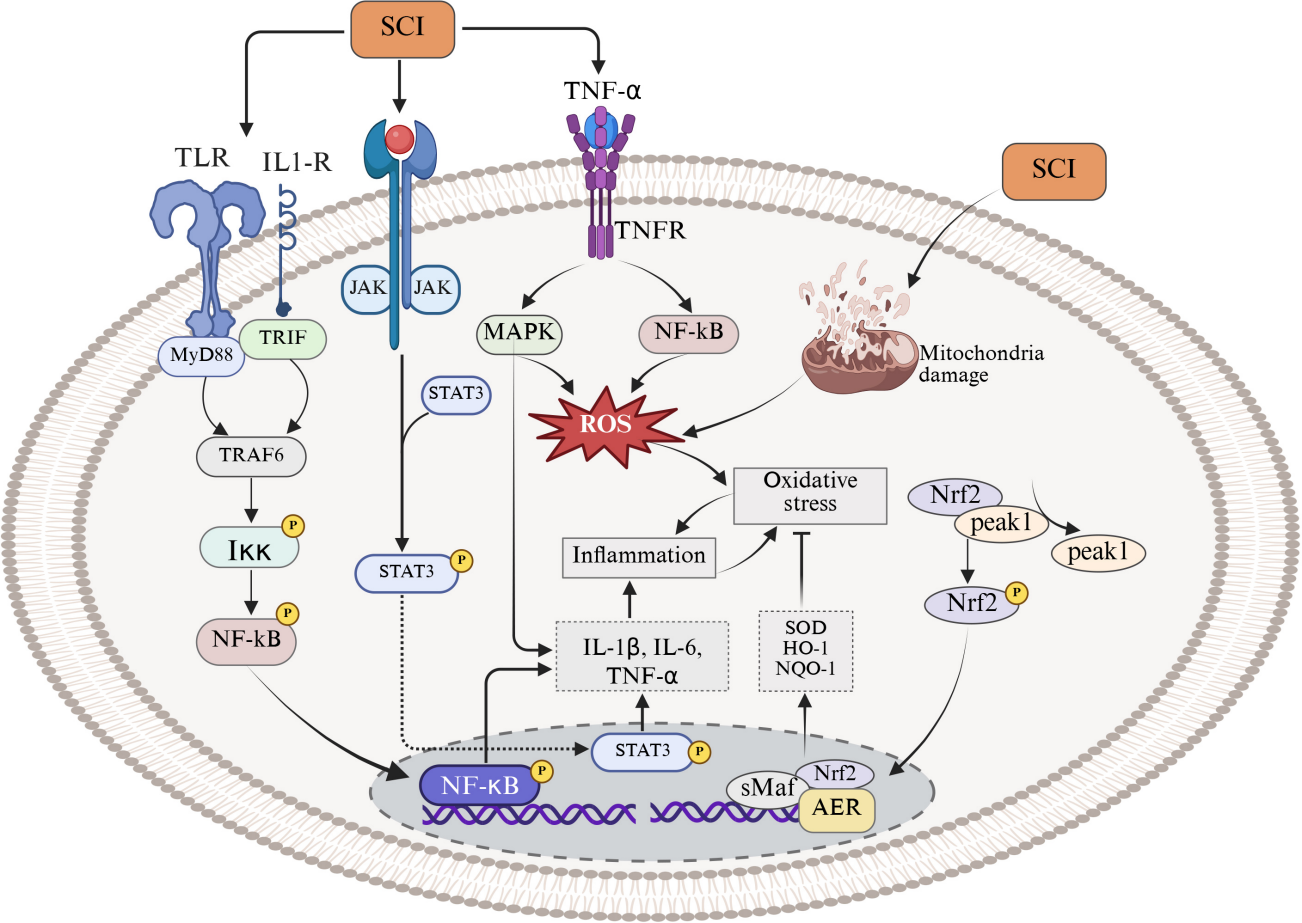
Signaling pathways related to the regulation of the inflammatory microenvironment after participating in tSCI.

MAPK is a group of evolutionarily highly conserved serine/threonine kinases and is one of the core regulatory pathways in the eukaryotic signal transduction network (196). This pathway can respond to different stimuli, link extracellular signals with fundamental cellular processes, and coordinate cellular responses and intercellular signaling (197, 198), thereby regulating cell growth, cell proliferation, cell differentiation, apoptosis, and stress responses (199, 200). After tSCI, the activation of MAPK is primarily triggered by acute tissue damage. Mechanical injury leads to the accumulation of a large number of DAMPs, pro-inflammatory cytokines (such as TNF-α, IL-1β, IL-6), and cell debris in the core injury area. These molecules can bind to and activate pattern recognition receptors (such as Toll-like receptors, TLRs) on the surface of immune cells and glial cells, subsequently triggering the phosphorylation cascade of p38, ERK, and JNK (201, 202). Once MAPK is phosphorylated and activated, it can initiate downstream transcriptional programs in microglia, infiltrating macrophages, and astrocytes. This activation induces pro-inflammatory transcription factors to enter the nucleus, which then significantly upregulate pro-inflammatory cytokines such as TNF-α, IL-1β, IL-6, as well as chemokines like CCL2 and CXCL1. These mediators recruit more peripheral monocytes/macrophages and T cells into the injury area and maintain the activated state of glial cells. Consequently, local inflammation is amplified, the BSCB is disrupted, neuronal death is promoted, and the range of secondary tissue damage expands (203–206). *In vitro* cell experiments have shown that inhibiting the phosphorylation of the MAPK pathway reduces the activation and translocation of inflammation-related transcription factors and decreases the expression of pro-inflammatory cytokines and chemokines both *in vitro* and *in vivo*, thereby alleviating inflammatory damage (207–209). Furthermore, not only does MAPK signaling drive inflammation during the acute phase, but it is also critical in the maintenance of neuropathic pain during the subacute to chronic stages (210, 211). tSCI leads to the sustained activation of microglia and astrocytes in the dorsal horn of the spinal cord. These glial cells, through sustained activation of MAPK (especially p38 and ERK), release pro-inflammatory cytokines and chemokines, which enhance the excitability of local synaptic transmission and upregulate pain signaling pathways, thereby inducing and maintaining central sensitization, hyperalgesia, and chronic neuropathic pain (212, 213). The activation of the MAPK signaling pathway has been confirmed to trigger neuropathic pain in a rat model of chronic pain after tSCI. Inhibiting the activity of this pathway can effectively suppress the expression of inflammatory factors in lipopolysaccharide-induced microglia, thereby alleviating symptoms associated with neuropathic pain (214, 215) (**Figure 3**).

## 5 Methods for controlling or eliminating the inflammatory microenvironment in tSCI

**Figure 4:**
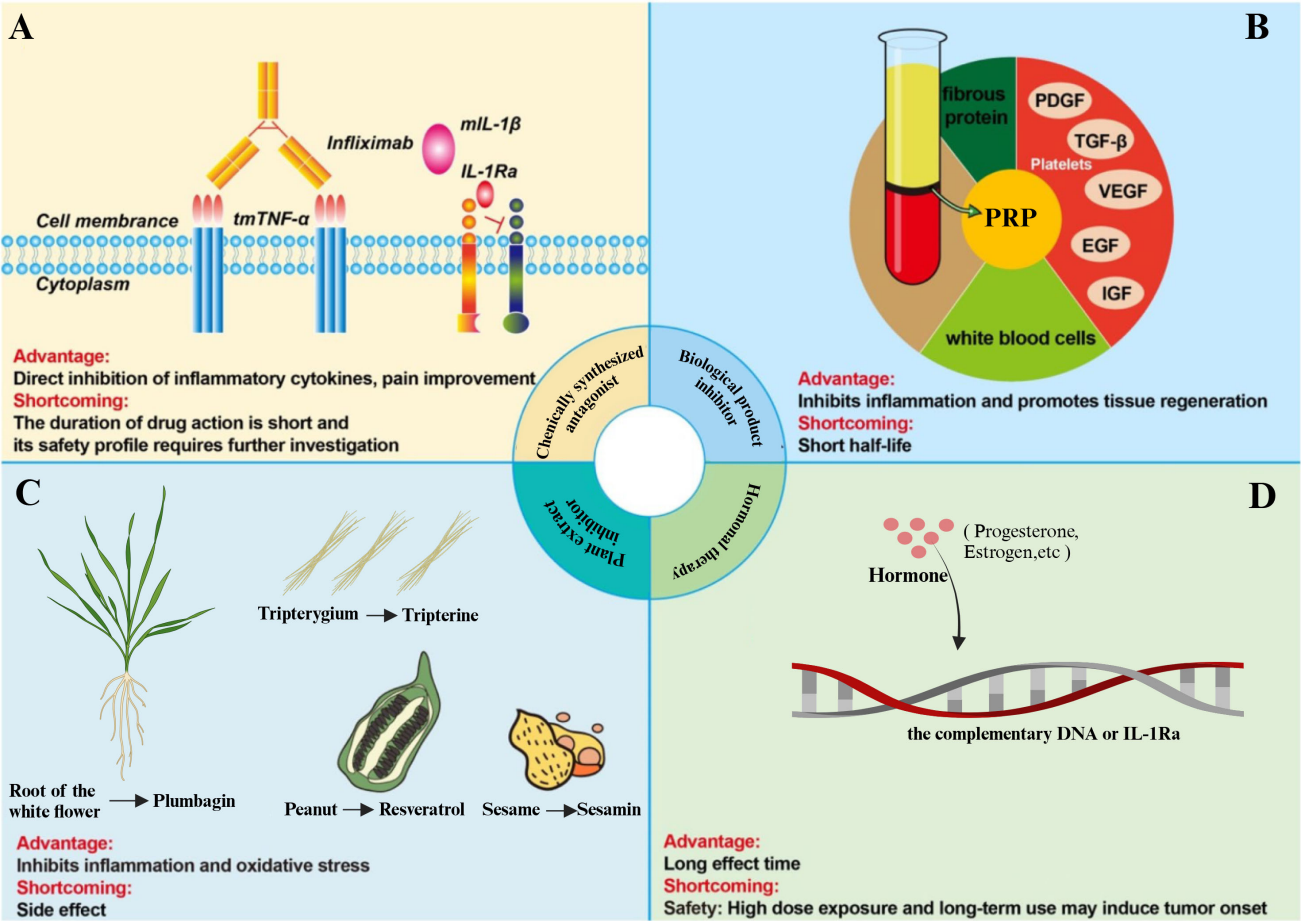
Methods to control or eliminate the inflammatory microenvironment of tSCI. **(A)** The TNF-α inhibitor, Infliximab, inhibits the binding of TNF-α to its receptor, thereby rendering TNF-α biologically inactive in triggering inflammatory pathways. The IL-1 inhibitor, IL-1Ra, binds to IL-1R, blocking downstream inflammatory signaling. **(B)** The biological product platelet-rich plasma (PRP) inhibits inflammation and promotes tissue regeneration. **(C)** Various plant extracts can attenuate the progression of tSCI by inhibiting inflammation and oxidative stress. **(D)** Hormonal therapy, such as corticosteroids, modulates the inflammatory response in tSCI by inhibiting key inflammatory pathways.

The highly complex local microenvironment following tSCI, particularly the persistent inflammatory milieu, amplifies secondary tissue damage, inhibits axon regeneration, and disrupts neural circuit reconstruction. These effects collectively hinder neuronal regeneration and functional recovery. Various anti-inflammatory strategies aim to modulate this inflammatory milieu by reducing inflammation at the injury site, regulating the balance between pro-inflammatory and anti-inflammatory responses, subsequently preventing further tissue damage, and promoting neuronal tissue repair and axonal regeneration. This section outlines several therapeutic approaches designed to modulate the inflammatory environment after tSCI (**Figure 4**).

### 5.1 Chemical synthesis antagonist

TNF-α inhibitors and IL-1 inhibitors are widely used for other inflammation-related diseases (such as rheumatoid arthritis) and have been proposed as potential therapeutic strategies for post-inflammatory regulation after tSCI. However, they are currently still mainly in the experimental and exploratory stages, with no clear evidence supporting their routine clinical applications in tSCI patients (216–218). Focusing on TNF-α inhibitors, Infliximab and Adalimumab are both monoclonal antibodies capable of neutralizing TNF-α activity. Infliximab can block the TNF-α/TNFR signaling pathway by specifically binding to TNF-α, thereby reducing the intensity of pro-inflammatory responses (219). In rat and rabbit models of tSCI, the application of Infliximab (a TNF-α neutralizing antibody) can inhibit TNF-α-mediated inflammatory vascular responses; reduce vascular permeability in the injured area and secondary edema; and significantly decrease neuronal loss (220, 221). In addition, Infliximab can reduce neuronal apoptosis by downregulating the expression of caspase-3, promoting the survival and regeneration of nerve cells, thus exerting neuroprotective effects (222).

Adalimumab (ADM) is a humanized monoclonal antibody whose unique structure can reduce immunogenicity and improve the safety of treatment. ADM can effectively inhibit TNF-α when used alone and can also be combined with other drugs (such as erythropoietin) to synergistically inhibit the M1 polarization of microglia, thereby alleviating inflammatory responses (223). In the tSCI rat model, the use of ADM combined with erythropoietin treatment can inhibit neuroinflammation and apoptosis mediated by microglial M1 polarization, thereby improving recovery from tSCI (224). Furthermore, Adalimumab reduces the release of pro-inflammatory factors by regulating the NF-κB signaling pathway, thereby enhancing the anti-inflammatory environment and promoting functional recovery of the spinal cord (225).

Interleukin-1 receptor antagonist (IL-1RA) is a molecule that antagonizes IL-1α and IL-1β by competitively binding to the IL-1 receptor (IL-1R), thereby preventing IL-1-mediated downstream inflammatory signal transduction and exerting anti-inflammatory effects (226). In the tSCI animal model, peripheral systemic administration of IL-1RA can weaken the excessive inflammatory reaction during the acute phase—including the elevation of pro-inflammatory factors, such as TNF-α and IL-1β—and reduce the infiltration of neutrophils, monocyte-derived macrophages, and T cells into the injury site, thus improving motor function recovery (227). Moreover, the abnormally high expression of IL-1 after tSCI is closely related to the amplification of local tissue damage. IL-1RA treatment can significantly reduce the level of IL-1β in the injured area, which alleviates secondary damage to neurons and glial cells (228). IL-1RA can also inhibit the activation of the NLRP3 inflammasome, thereby reducing caspase-1-mediated pyroptosis. This effect alleviates cell death and functional impairment caused by spinal cord injury (229). Additionally, the anti-inflammatory mechanism of IL-1RA includes regulating the phenotypic conversion of macrophages and microglia, promoting the proliferation of M2 macrophages, and thereby forming an anti-inflammatory microenvironment that is conducive to nerve regeneration (230).

COX-2 is another target for modulating the inflammatory microenvironment, as it regulates the synthesis of PGE2 in this environment (231). In the tSCI rat model, intraperitoneal injection of COX-2 inhibitors can alleviate the inflammatory response by inhibiting neutrophil infiltration and reducing the synthesis of prostaglandins and free radicals (232). Furthermore, in tSCI animal models, the selective COX-2 inhibitor meloxicam has been found to inhibit COX-2-mediated PGE2 production, significantly reducing neutrophil infiltration into the injury site, alleviating local tissue edema and secondary mechanical compression, and promoting motor function recovery (233). Therefore, COX-2 inhibitors, as an adjunctive therapeutic approach to standard treatments, have significant potential in the modulation of inflammation after tSCI and may become a new strategy for future clinical treatment.

### 5.2 Biological product inhibitors

The main components of platelet-rich plasma (PRP) are platelets, leukocytes, and plasma. Platelets are the core of PRP because they contain high concentrations of various growth factors that play an important role in promoting tissue repair and regeneration (234). PRP contains growth factors such as platelet-derived growth factor, TGF-β, epidermal growth factor, insulin-like growth factor 1, and VEGF. It also plays a significant role in regulating the inflammatory microenvironment after spinal cord injury. In a rat tSCI model, PRP treatment can downregulate the transcription levels of TNF-α and IL-6, primarily by blocking the TLR4/MyD88 pathway, thereby inhibiting neuroinflammatory responses (235, 236). Furthermore, in an experimental spinal cord contusion model, local injection of PRP can enhance the expression of tight junction proteins, such as ZO-1, alleviate the inflammatory environment in the damaged area, and repair the blood-spinal cord barrier. These effects improve the microenvironment after spinal cord injury, contributing to the recovery of neurological function and nerve regeneration (237).

Mesenchymal stem cells (MSCs) are multipotent stem cells derived from the mesoderm, possessing self-renewal and multi-lineage differentiation potential. MSCs are regarded as multipotent progenitor “seed cells” that can inhibit local inflammation and apoptosis. They also stimulate the regeneration and differentiation of resident tissue progenitor cells by secreting soluble growth factors. In the context of tSCI, MSCs can be induced to differentiate into neuron-like cells, providing a potential cellular source for nerve regeneration (238–240). In rat spinal cord contusion and compressive tSCI models, transplanted MSCs reduce the inflammatory response at the injury site by secreting anti-inflammatory cytokines and inhibiting pro-inflammatory cytokines, such as TNF, IFN-γ, and IL-6, thereby playing a crucial role in promoting tissue repair (241–243). Additionally, in a mouse contusion tSCI model, transplanting neural stem cells (NSCs) to the injury site can reduce neutrophil infiltration and regulate macrophage activation by inhibiting the pro-inflammatory M1 macrophage phenotype. NSCs lower the mRNA levels of inflammatory cytokines, including TNF-α, IL-1β, IL-6, and IL-12. They also inhibit the activation of bone marrow-derived macrophages, reduce the release of cytokines such as TNF-α and IL-1β, and improve functional recovery after tSCI (244).

### 5.3 Plant extract inhibitors

In recent years, a large number of rodent model studies have utilized plant-derived components to alleviate the inflammatory microenvironment of tSCI (245, 246). For example, compounds derived from plants such as curcumin, geniposide, sesamin, baicalin, rutin, and resveratrol have been shown to promote the recovery of neurological function after injury in tSCI models by inhibiting the inflammatory response induced by IL-1β or TNF-α (247).

Geniposide is an active ingredient of gardenia, a traditional Chinese medicinal herb, which exhibits various pharmacological effects, such as anti-inflammatory, antioxidant, and anti-apoptotic properties (248, 249). In tSCI cell models, geniposide reduces the expression and release of IL-1β, IL-6, IL-8, and TNF-α induced by LPS (250). In tSCI rat models, geniposide treatment significantly inhibited the IKKs/NF-κB signaling pathway, reduced neutrophil infiltration at the injury site, and significantly downregulated TNF-α, IL-1β, and IL-6, thereby alleviating spinal cord damage caused by the inflammatory response (251, 252). Moreover, geniposide reduces inflammatory factor expression by inhibiting the activation of the NF-κB signaling pathway, thereby exerting its anti-inflammatory effects (253). Similarly, sesamin is a component of sesame oil with anti-inflammatory and neuroprotective effects (254). In rat tSCI models, sesamin exerts its anti-inflammatory effects and promotes the recovery of motor function by inhibiting NF-κB activation and upregulating AMPK signaling (255). Furthermore, sesamin promotes cellular autophagy by activating the AMPK signaling pathway, which plays a key role in neuroprotection. The activation of AMPK helps regulate cell metabolism and inflammatory response, allowing nerve cells to better recover function when injured (256, 257). Baicalin, extracted from the roots of Scutellaria baicalensis, exhibits multiple biological activities, such as anti-inflammatory and analgesic effects (258). In tSCI rat models, baicalin can improve neurological function after spinal cord injury by activating Nrf-2, inhibiting the activity of NF-κB, and alleviating cellular inflammatory response and oxidative stress (259). In addition to the aforementioned plant-derived compounds, rutin (260), mangiferin (261), tripterine (262), and resveratrol glycoside (245) can also reduce NLRP3 expression in tSCI animal models, thereby decreasing the release of pro-inflammatory factors such as IL-1β and IL-18, effectively inhibiting the inflammatory response and promoting the recovery of neurological function.

### 5.4 Hormone therapy

Estrogens, as an important sex hormone, mainly exert their biological effects by activating estrogen receptors (ERα and ERβ). In the regulation of inflammation after tSCI, estrogens can inhibit the activation of inflammatory signaling pathways such as NF-κB. This inhibition downregulates the gene expression of TNF-α and iNOS and reduces local inflammation levels. In a rat spinal cord injury contusion model, treatment with 17-β estradiol (E2) significantly reduced the levels of IL-1β and IL-18 in spinal cord tissue, while improving neuronal survival and enhancing the recovery of hind limb motor function (263). Furthermore, estrogens have also been found to alleviate the neuroinflammatory response after tSCI by inhibiting the activity of the NLRP3 inflammasome, which may provide a new explanation for the neuroprotective effects of estrogens in tSCI (264, 265). Progestogens exert their biological effects by binding to progesterone receptors (PR); in tSCI, exogenous progestogen administration can inhibit the activation of the NLRP3 inflammasome, reducing the release of IL-1β and MCP-1 in the injured area, accompanied by a reduction in local inflammatory infiltration (266). In a mouse tSCI model, systemic administration of progestogens significantly alleviated spinal cord edema and oxidative stress levels and improved motor function scores by reducing neuronal apoptosis, suggesting that progestogens have potential therapeutic value in promoting neuronal survival and functional recovery (267). Additionally, progestogens demonstrate significant effects in regulating the immune response after tSCI, further promoting the recovery of neurological function by modulating mechanisms that protect neurons (268). Besides estrogens and progestogens, research on corticosteroids and androgens in the regulation of inflammation in tSCI has also attracted considerable research interest. Corticosteroids exhibit typical anti-inflammatory effects in acute tSCI animal models, inhibiting the release of pro-inflammatory cytokines and alleviating local tissue inflammation and secondary injury; however, their long-term use or high-dose application may lead to side effects such as immunosuppression and osteoporosis (269). Androgens are believed to have potential neuroprotective effects in promoting nerve regeneration and repair, with early experimental administration in rodent tSCI models showing that androgens may promote axonal preservation/regeneration and subsequent improvement in motor function (270). Nevertheless, existing evidence remains insufficient regarding the specific mechanisms of hormonal intervention, optimal administration windows, dosage ranges, and individualized medication strategies, especially given the limited research foundation for translating findings from rodent models to clinical patients (271).

## 6 Future prospects and conclusions

Traumatic spinal cord injury is a common neurotrauma disease that can lead to disability and negatively impact quality of life. Inflammation is a relevant process in the pathophysiology of tSCI, and the inflammatory microenvironment is considered an important factor in the occurrence and development of tSCI, where various inflammatory cells and mediators play a crucial role. These cell types and inflammatory cytokines trigger inflammatory cascade reactions through multiple mechanisms, accelerating the deterioration of the inflammatory microenvironment, which in turn leads to further damage to the spinal cord. Regulating the inflammatory microenvironment can effectively inhibit the pathological progression of spinal cord injury, thereby alleviating tSCI symptoms and improving prognosis. Therefore, regulating the inflammatory microenvironment may be an effective approach to treating tSCI.

Despite the progress made in this research area, several limitations remain. In terms of mechanism research, although key mechanisms such as the activation and infiltration of immune cells—such as microglia, macrophages, and neutrophils—the release of inflammatory factors, and inflammatory cascade reactions have been thoroughly explored, the complex interactions between various cells and molecules in the inflammatory microenvironment have not been fully clarified. The functions and mechanisms of newly discovered cell subpopulations and molecules, such as recently identified immune cell subsets and novel cytokines, need further exploration. Additionally, the interaction mechanisms between the inflammatory microenvironment and neuroregenerative cells, including neural stem cells and neural progenitor cells, require in-depth study. Regarding intervention strategies, most existing treatment methods remain in the basic research or clinical trial stages and have not yet resulted in mature and effective clinical treatment plans. The safety, efficacy, and long-term effects of various treatment methods still need further validation. Moreover, combination therapy strategies—such as the concurrent or sequential use of different treatments—also need to be studied in depth to achieve optimal therapeutic effects. In clinical case analyses, the sample sizes are relatively small, which may not fully reflect the situation of tSCI patients. Additionally, the research durations are short, necessitating further observation and study on the long-term effects of intervention strategies.

In the future, mechanistic studies of the inflammatory microenvironment in spinal cord injury and the development of therapeutic strategies have broad prospects. With the continuous advancement of biotechnology, such as single-cell sequencing and multi-omics analysis, it will be possible to reveal the functions and interaction mechanisms of various cells and molecules in the inflammatory microenvironment more deeply. Combined with gene editing technologies, such as CRISPR/Cas9, research will further explore the role of specific genes in the inflammatory microenvironment and nerve repair, providing a theoretical basis for developing new therapeutic targets. Regarding therapeutic strategies, it is necessary to further optimize existing treatment methods to improve efficacy and safety. Research on the combined application of different treatment methods should be strengthened to explore the best combination therapy plans for synergistic effects. Additionally, there is a need to develop new treatment methods and technologies, such as personalized therapy strategies based on artificial intelligence and the application of nanotechnology in both drug delivery and cell therapy. In clinical research, the sample size should be expanded, and multicenter, large-sample clinical trials should be conducted to further verify the effectiveness and safety of these interventional strategies. Strengthening long-term follow-up studies to assess the sustained efficacy and potential adverse reactions of therapeutic strategies is also crucial. Through continuous research and exploration, researchers are expected to reveal the mechanisms of the inflammatory microenvironment in spinal cord injury in depth, develop more effective therapeutic strategies, improve nerve function recovery in spinal cord injury patients, and enhance their quality of life.
